# Evaluation of neutrophil activation in autoimmune kidney diseases using blood heparin-binding protein: a pilot study

**DOI:** 10.3389/fmed.2026.1727663

**Published:** 2026-02-04

**Authors:** Zhen-jun Zhao, Xin-yue Shi, Hong-shan Chen, Hao-miao Zhang, Ying Gao, Ming-xuan Gao, Chen-xi Cai, Zi-rui Liu, Jun-ya Jia, Peng-cheng Xu

**Affiliations:** 1 Department of Nephrology, Kidney Disease Medical Center, General Hospital, National Key Clinical Specialty, Tianjin Key Medical Discipline, Tianjin Medical University, Tianjin, China; 2 Department of Rheumatology and Immunology, The Affiliated Hospital of Inner Mongolia Medical University, Hohhot, Inner Mongolia, China; 3 Department of Critical Care Medicine, Tianjin Medical University General Hospital, Tianjin, China

**Keywords:** anti-neutrophil cytoplasmic antibodies-associated vasculitis, autoimmune kidney diseases, focal segmental glomerulosclerosis, heparin-binding protein, IgA nephropathy, membranous nephropathy, minimal change disease, neutrophil

## Abstract

**Background:**

Currently, there is limited understanding about the activation of neutrophils in autoimmune kidney diseases. Heparin-binding protein (HBP) is a protein in neutrophil granules and has been widely used as a marker for infectious diseases. This study aims to explore the use of blood HBP to evaluate neutrophil activation in autoimmune kidney diseases.

**Methods:**

A total of 70 patients diagnosed with autoimmune kidney diseases were enrolled, including 20 with anti-neutrophil cytoplasmic antibodies-associated vasculitis (AAV), 17 with membranous nephropathy (MN), 17 with IgA nephropathy (IgAN), and 16 with minimal change disease (MCD)/focal segmental glomerulosclerosis (FSGS). The control group consisted of 18 patients with sepsis, 20 patients on maintenance hemodialysis, and 20 healthy volunteers. Clinical and laboratory indicators were collected, and the blood HBP were measured.

**Results:**

The blood HBP were significantly elevated in all kinds of autoimmune kidney disease, with the highest levels in AAV and the lowest in MCD/FSGS. Unlike blood NGAL, blood HBP was not affected by estimated glomerular filtration rate (eGFR). Blood HBP was independently correlated with blood neutrophil counts, and its sensitivity was higher than that of blood NGAL, neutrophil counts, and C-reactive protein. In AAV, blood HBP was associated with hematuria, and its levels significantly decreased after remission. In MN and MCD/FSGS, elevated HBP was associated with higher levels of proteinuria. There was a positive correlation between blood HBP and urine HBP in patients with autoimmune kidney diseases.

**Conclusion:**

Blood HBP is a valuable and eGFR-independent marker of neutrophil activation. This study preliminarily reveals that neutrophil activation is widespread in various autoimmune kidney diseases and may be involved in the pathogenesis of these diseases.

## 1 Introduction

Autoimmune kidney diseases, such as anti-neutrophil cytoplasmic AAV, MN, IgAN, and MCD/FSGS, are the main causes of end-stage renal disease in clinical practice. Their common pathogenesis lies in the inflammatory response triggered by abnormal activation of the immune system. Neutrophils, as the core effector cells of innate immunity, play different roles in various autoimmune kidney diseases (1). In AAV, the abnormal activation of neutrophils and the formation of neutrophil extracellular traps (NETs) are common and crucial (2). In IgAN, although some experimental researches suggest that neutrophils may be involved in the pathogenesis, there are still many controversies regarding this issue due to the lack of evidence in clinical practice. In MN and MCD/FSGS, the understanding of whether neutrophil activation is involved in the disease pathogenesis is even more limited. Even in AAV, which has been considered as a classic “neutrophil-mediated disease,” there is currently a lack of sensitive clinical methods to assess the degree of neutrophil activation.

Heparin-binding protein (HBP), also known as azurocidin (AZU) and cationic antimicrobial protein of 3,700 (CAP37), is a multifunctional single-chain glycoprotein with bactericidal and chemotactic properties, mainly present in the secretory vesicles and azurophilic granules (also known as metachromatic granules) of neutrophils, and plays a role in the pathophysiology of endothelial dysfunction during inflammation (3). The secretory vesicles are the main compartment for the release of HBP, and activated azurophilic granules also release a small amount of HBP. In infectious diseases, especially sepsis, blood HBP levels often increase significantly, and thus HBP level detection has been widely used as a marker for diagnosing infectious diseases (4, 5). Currently, there are few studies on HBP in non-infectious diseases. In this study, we detected the levels of HBP in the peripheral blood of various autoimmune kidney diseases and analyzed its value and clinical significance as a marker for assessing neutrophil activation in these diseases.

## 2 Methods

### 2.1 Research subjects

This study selected 70 patients diagnosed as autoimmune kidney diseases in the Department of Nephrology of Tianjin Medical University General Hospital from April 2024 to September 2025, including 20 patients with AAV, 17 patients with primary MN, 17 patients with IgAN, and 16 patients with MCD/FSGS. We also selected 18 patients with sepsis from the Department of Critical Care Medicine of Tianjin Medical University General Hospital during the same period as positive controls, and 20 healthy volunteers as negative controls. Additionally, to analyze the impact of renal function impairment on blood HBP, we selected 20 patients undergoing maintenance hemodialysis at the Hemodialysis Center of Tianjin Medical University General Hospital. This study was performed with the approval of the Ethics Committee of Tianjin Medical University General Hospital (IRB2025-KY-270). All patients signed informed consent forms.

### 2.2 Inclusion criteria

All subjects included in this study were aged 18 to 80 years old. All patients with autoimmune kidney diseases were newly diagnosed and had not received any Immunosuppressive therapy. Patients with renal injury induced by AAV must meet the AAV classification criteria jointly formulated by the American College of Rheumatology (ACR) and the European League Against Rheumatism (EULAR) in 2022 (6, 7), and have one of the following conditions: (1) glomerular hematuria, (2) proteinuria, (3) renal insufficiency, (4) kidney biopsy confirming pauci-immune complex focal necrotizing glomerulonephritis. The diagnosis of MN must simultaneously meet the following conditions: (1) proteinuria as the main clinical manifestation, (2) positive serum phospholipase A2 receptor antibody, or kidney biopsy confirming primary MN. The diagnosis of IgAN must simultaneously meet the following conditions: (1) clinical manifestations of hematuria and/or proteinuria, (2) kidney biopsy showing IgA-dominant immune complex deposition in the glomerular mesangial area. The diagnosis of MCD/FSGS must simultaneously meet the following conditions: (1) nephrotic syndrome-level proteinuria, (2) light microscopy of kidney biopsy showing normal glomerular morphology or partial glomeruli and some glomerular capillary loops undergoing sclerosis, and electron microscopy showing diffuse podocyte fusion without electron-dense deposits. The inclusion of sepsis patients must simultaneously meet the following conditions: (1) presence of clinically suspected or microbiologically confirmed infection, (2) confirmed sepsis according to the International Consensus Definitions for Sepsis and Septic Shock (Sepsis-3), that is, an acute increase of ≥2 points in the Sequential Organ Failure Assessment Score due to infection (8). Patients in maintenance hemodialysis refer to those clinically diagnosed with chronic end-stage renal disease and have been in the hemodialysis treatment stage for ≥3 months, with regular dialysis frequency (2–3 times per week).

### 2.3 Exclusion criteria

(1) AAV patients with concurrent lupus nephritis or anti-glomerular basement membrane disease. (2) Secondary MN due to other diseases (such as hepatitis B virus infection, malignant neoplasm, lupus). (3) Secondary IgAN due to other diseases (such as Henoch-Schönlein purpura, chronic liver disease). (4) Secondary FSGS (including those with clear causes and those without clear causes but insensitive to glucocorticoid treatment). (5) Patients in maintenance hemodialysis had concurrent active infection, malignant tumors, autoimmune diseases, etc.

### 2.4 Clinical indicators collection

This study collected clinical indicators of patients with autoimmune kidney diseases, including basic demographic characteristics (gender, age), disease diagnosis information, Birmingham Vasculitis Activity Score (BVAS) score of AAV, blood cell analysis (including various cell counts), laboratory indicators related to kidney injury (serum creatinine, 24 h urine protein, 24 h urine microalbumin, urine red blood cells), and C-reactive protein (CRP). The serum neutrophil gelatinase-associated lipocalin (NGAL) was quantified using Immunolatex turbidimetry, employing a commercial kit (Catalog Number: R21220102, Beijing Sjodax Biotech, Changping, Beijing, China) in accordance with the provided instructions. The eGFR was calculated using the modified MDRD formula (9).

### 2.5 Detection of blood HBP

The detection of blood HBP is carried out using the magnetic particle chemiluminescence method (Hunan Yonghe Sunshine Biotechnology, China, registration number: Xiang Medical Device Registration 20212401968). All patients had their blood drawn and anticoagulated with sodium citrate (dilution ratio 1 : 9). The two monoclonal antibodies against HBP in the kit are, respectively, labeled with magnetic beads and pyridinoline. They can react with the analyte in the sample to form a double-antibody sandwich complex of pyridinoline-labeled anti-HBP antibody-1-HBP-magnetic bead-labeled anti-HBP antibody-2. Then, a strong magnetic field is applied to adsorb the complex in the reaction container. The pyridinoline in the complex generates chemiluminescence under the action of drugs in the fully automatic immunoassay system. The content of HBP in the sample to be tested is read from the standard curve. The detection is performed using the Shine i2910 fully automatic chemiluminescence immunoassay analyzer from Shenzhen Dikai Biotechnology. The detection range of the kit is 6 ng/mL to 300 ng/mL. If the content is higher than the upper limit of detection, the sample is diluted at a ratio of 1:2. The final result of the clinical sample is reported based on the measured concentration after dilution and the dilution factor (3 times). The coefficient of variation (CV) of repeatability is ≤10%. The CV of inter-batch difference is ≤15%.

### 2.6 Detection of blood myeloperoxidase (MPO)

Blood MPO concentration was tested by a Human MPO ELISA Kit (KE00353, Proteintech Group, Inc. Wuhan Sanying, Wuhan, Hubei, China). An antibody specific for human MPO had been pre-coated onto the microplate wells. During incubation, human MPO protein present in the blood samples (1:200) was captured by the immobilized antibody. After thorough washing to remove unbound components, a horseradish peroxidase -conjugated antibody specific for human MPO was added to detect the captured antigen. For signal generation, tetramethylbenzidine (TMB) substrate was introduced. The resulting color intensity was measured at 450 nm with a correction wavelength of 630 nm. The Sensitivity was 10.1 pg/mL.

### 2.7 Detection of urinary HBP

The level of urinary HBP was determined by an ELISA kit (JL15210, Jonlnbio Industrial Co., Ltd., Shanghai, China). To the microplate wells pre-coated with HBP capture antibody, diluted urine (1:20), standards and biotin-labeled detection antibody were added successively. After incubation and washing, HRP conjugate was added. The color was developed with TMB substrate and the absorbance was measured at 450 nm with an enzyme immunoassay reader. The sample concentration was calculated. The sensitivity was 38.66 pg/mL.

### 2.8 Statistics

Normally distributed data were presented as mean ± standard deviation, while non-normally distributed data were expressed as median. The Kruskal–Wallis *H* test, a non-parametric test, was used to compare the HBP levels among multiple disease groups. One-way analysis of variance (ANOVA) was employed to compare the ages of patients across different autoimmune kidney disease groups. For the remaining non-normally distributed variables, non-parametric tests were utilized for intergroup comparisons. Spearman correlation and partial correlation were employed to analyze the relationships between different variables and eGFR levels as well as between different variables and NC levels. Chi-square tests were conducted to compare the percentages of elevated blood HBP, NC, and CRP in the overall autoimmune kidney disease population and in each disease subgroup. Paired sample *T*-tests were used to statistically analyze the differences in blood HBP levels between the active and remission phases of AAV. Multivariable linear regression models were used to analyze factors influencing blood HBP in autoimmune kidney diseases. All *p*-values were two-tailed, and values <0.05 were considered statistically significant. Statistical analyses were performed using SPSS (version 26; IBM SPSS, Chicago, IL, United States).

## 3 Results

### 3.1 The levels of blood HBP in all kinds of autoimmune kidney diseases were significantly elevated

**Figure 1:**
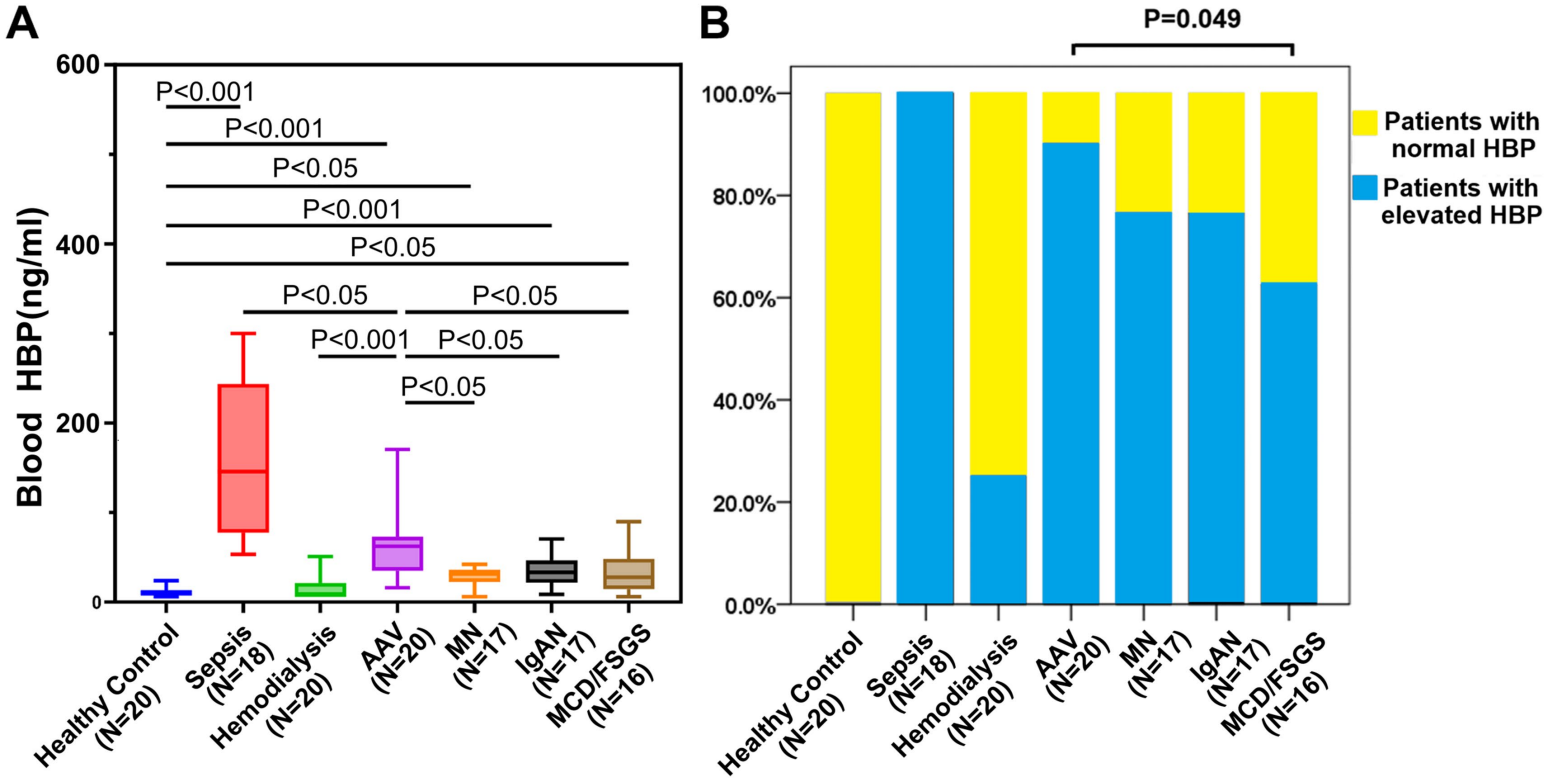
Comparison of blood HBP levels among different groups. **(A)** Comparison of median blood HBP levels between healthy control, patients with sepsis, patients with maintenance hemodialysis, and patients with different autoimmune kidney diseases, revealing that patients with sepsis exhibited the highest blood HBP levels. **(B)** The normal range of blood HBP was established based on the HBP levels in healthy control, followed by a comparison of the proportion of elevated blood HBP among patients with different diseases.

The clinical characteristics of all patients with autoimmune diseases are presented in Table 1. First, we measured the blood HBP in 20 healthy controls, with an average of 11.18 ± 4.67 ng/mL. The upper limit of normal was set at 20.52 ng/mL, which was two times the standard deviation. Since the blood HBP levels in the other groups were not normally distributed, the median was used to describe them. As shown in Figure 1A, the blood HBP level was the highest in sepsis patients (median: 145.75 ng/mL), and was significantly higher than that in any other group. The median blood HBP levels in patients with autoimmune kidney diseases were all higher than those in the healthy control group, and the differences were statistically significant (*p* < 0.05). The median blood HBP level in patients with autoimmune kidney diseases was also higher than that in patients on maintenance hemodialysis (*p* < 0.05). Among different kinds of autoimmune kidney diseases, the median blood HBP level in AAV was higher than that in MN, IgAN, and MCD/FSGS, and the differences were statistically significant (all *p* < 0.05). There was no statistically significant difference in the median blood HBP levels among the MN, IgAN, and MCD/FSGS (*p* > 0.05).

**Table 1:** Clinical characteristics of patients with autoimmune diseases.

Factors	AAV	MN	IgAN	MCD/FSGS	*p*-value
	*N* = 20	*N* = 17	*N* = 17	*N* = 16	
Age (years)	66.6 ± 58.86	53.41 ± 12.73	44.82 ± 12.86	45.13 ± 17.46	<0.001
Male/female	10/10	12/5	5/12	8/8	0.124
Scr (μmol/L)	354.50 (143.25, 626.50)	72.00 (59.50, 83.00)	75.00 (66.50, 95.50)	79.00 (64.00, 120.75)	<0.001
Proteinuria (mg/24 h)	868.50 (296.25, 4096.75)	3037.50 (1867.45, 7665.00)	1155.00 (369.00, 2807.00)	4980.00 (2336.00, 6912.00)	<0.001
Hematuria (cells/μL)	188.00 (19.93, 615.95)	80.50 (18.98, 132.53)	174.50 (46.95, 251.95)	16.05 (6.68, 100.45)	0.006

AAV, ANCA-associated vasculitis; FSGS, focal segmental glomerulosclerosis; IgAN, IgA nephropathy; MCD, minimal change disease; MN, membranous nephropathy; Scr, serum creatinine. *p*-value <0.05 was considered statistically significant.

We also compared the proportion of patients with elevated blood HBP in each group. As shown in Figure 1B, all 18 sepsis patients had elevated blood HBP (100%), 5 out of 20 patients in maintenance hemodialysis had elevated blood HBP (25%), 18 out of 20 AAV patients had elevated blood HBP (90%), 13 out of 17 MN patients had elevated blood HBP (76.47%), 13 out of 17 IgAN patients had elevated blood HBP (76.47%), and 10 out of 16 MCD/FSGS patients had elevated blood HBP (62.5%). Among 70 patients with autoimmune kidney diseases, the proportion of AAV patients with elevated blood HBP was slightly higher than that of MCD/FSGS patients (*p* = 0.049), and there was no statistically significant difference among the other groups.

### 3.2 Blood MPO and NGAL are not significantly elevated in non-AAV autoimmune kidney diseases

**Figure 2:**
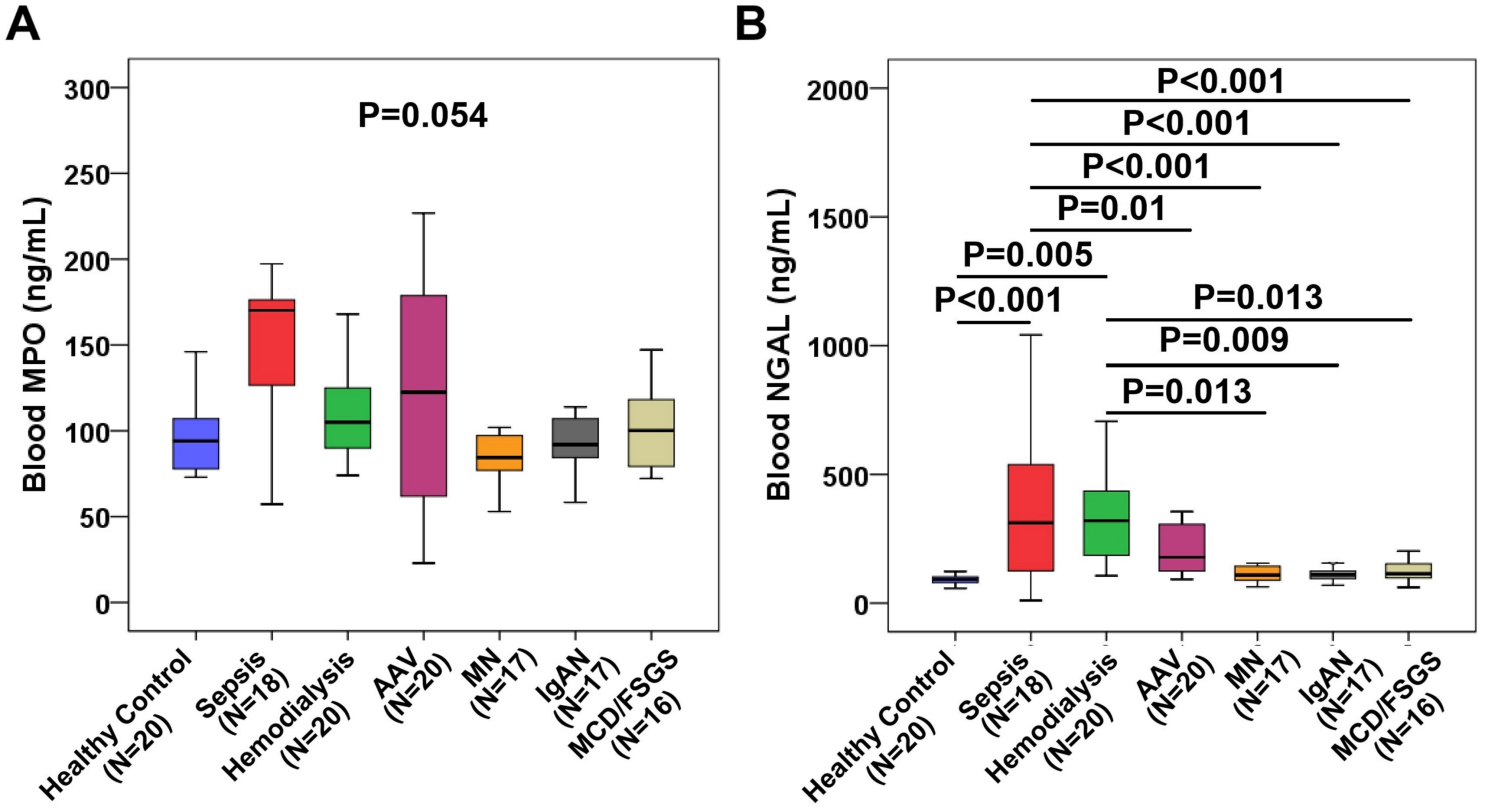
Comparison of blood MPO and NGAL among different disease groups. **(A)** Comparison of blood MPO among different groups. **(B)** Comparison of blood NGAL among different groups.

Given the multitude of neutrophil activation markers, we selected two additional markers, MPO and NGAL, for assessment across all patients with various diseases. As shown in Figure 2A, no statistically significant difference in blood MPO levels was observed among all the disease groups (*p* = 0.054). However, the highest blood MPO levels were noted in patients with sepsis and AAV. As shown in Figure 2B, patients with sepsis and those on maintenance hemodialysis exhibited the highest and comparable blood NGAL levels among all groups. While AAV patients tended to have higher blood NGAL levels than patients with other types of autoimmune kidney diseases and the normal control group, although the difference did not reach statistical significance.

### 3.3 Compared with blood NGAL, blood HBP levels are not affected by eGFR and are more sensitive

It is still unclear whether blood HBP is affected by renal function. First, we conducted a simple correlation analysis in 70 patients with autoimmune nephropathy to find which indicators were related to eGFR. As shown in Table 2, age, blood HBP levels, blood NGAL levels, peripheral blood neutrophil count (NC), and blood CRP levels were correlated with eGFR, among which blood HBP had a weak negative correlation with eGFR (*R* = −0.335, *p* = 0.005). However, in the partial correlation analysis, blood HBP was no longer correlated with eGFR, while blood NGAL levels still had an obvious negative correlation with eGFR. This indicates that blood HBP levels are not affected by eGFR levels, which is consistent with the result that the blood HBP levels of patients in maintenance hemodialysis are not different from those of normal control in the first part of the results. On the contrary, the clearance of blood NGAL is affected by renal function. Therefore, in patients with renal function impairment, blood NGAL levels cannot represent the degree of neutrophil activation.

**Table 2:** Correlation between eGFR and different inflammatory indicators.

Factors	Single-correlation			Partial-correlation		
	*R* value	95% CI	*p*-value	*R* value	95% CI	*p*-value
Age	−0.52	−0.677~−0.318	<0.001	−0.48	−0.641~−0.278	<0.001
Serum HBP	−0.335	−0.533~−0.101	0.005	−0.167	−0.449~0.116	0.224
Serum NGAL	−0.538	−0.695~−0.333	<0.001	−0.377	−0.554~−0.147	0.005
Neutrophil count	−0.245	−0.459~−0.004	0.041	0.11	−0.175~0.362	0.423
Serum CRP	−0.685	−0.8~−0.521	<0.001	−0.047	−0.248~0.187	0.735

HBP, Heparin-binding protein; NGAL, Neutrophil gelatinase-associated lipocalin; CRP, C-reactive protein; eGFR, Estimated glomerular filtration rate; CI, Confidence interval. *p*-value <0.05 was considered statistically significant.

**Figure 3:**
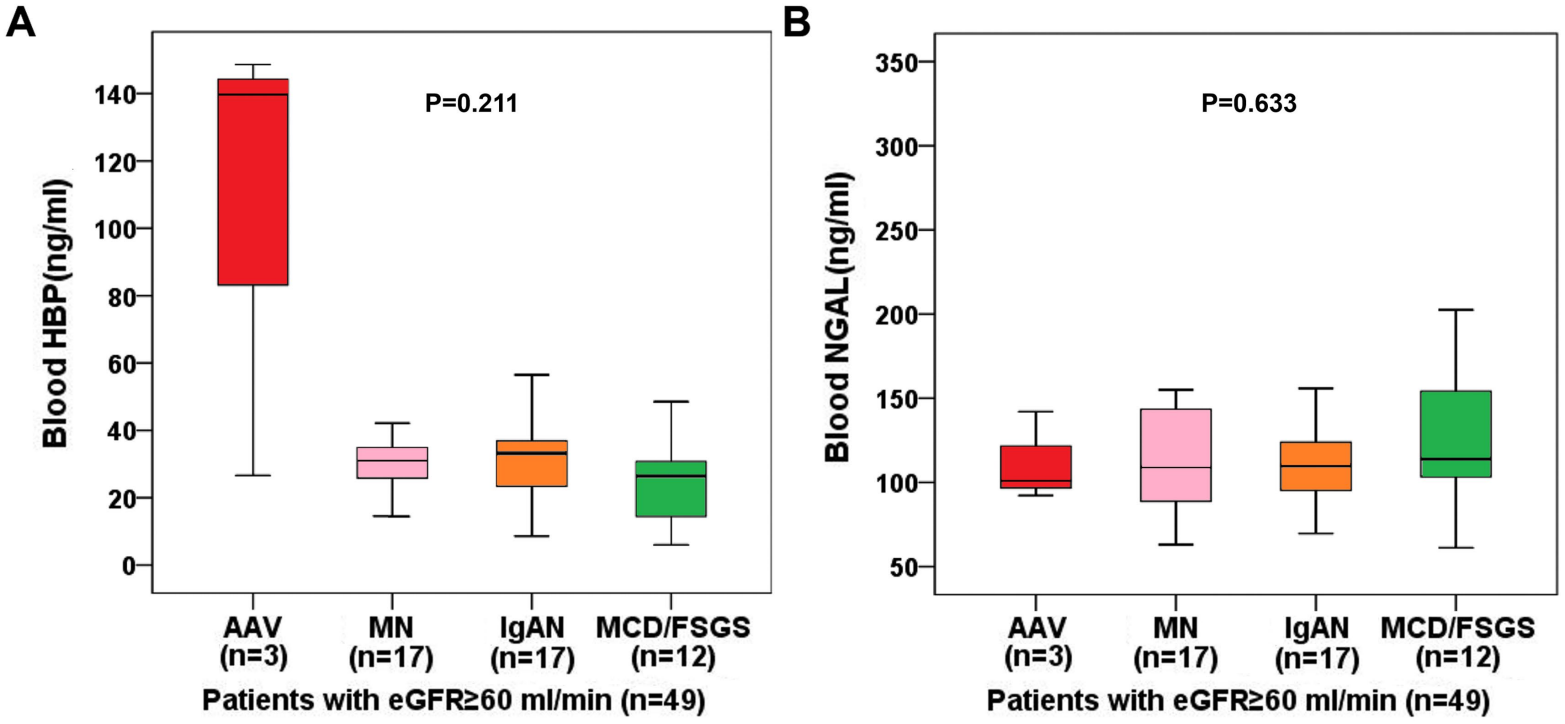
Among 70 patients with autoimmune kidney diseases, 49 patients with eGFR ≥60 mL/min were selected to compare the differences in blood HBP levels **(A)** and blood NGAL levels **(B)** among patients with different kinds of autoimmune kidney diseases.

As blood NGAL is influenced by eGFR, we selected 49 patients with eGFR ≥60 mL/min from 70 patients with autoimmune-related kidney diseases and compared the sensitivity of blood HBP and blood NGAL in representing neutrophil activation. Since most AAV patients have renal function impairment, only 3 AAV patients remained after excluding those with eGFR <60 mL/min, while there were 17 patients with MN, 17 with IgAN, and 12 with MCD/FSGS, respectively. Among the 49 patients with eGFR ≥60 mL/min, 36 had elevated blood HBP and only 3 had elevated blood NGAL, showing a significant statistical difference (*p* < 0.001). As shown in Figure 3, although the comparison did not reach statistical significance due to the small sample size, the blood HBP level in AAV still tended to be significantly higher than that in MN, IgAN, and MCD/FSGS (*p* = 0.211), while the blood NGAL level in AAV did not show a trend of being higher than the other three groups (*p* = 0.633). Therefore, in patients with normal renal function, blood NGAL is insufficient as a marker for representing neutrophil activation, even for AAV that has been fully proven to have the characteristics of neutrophil activation.

### 3.4 Blood HBP is correlated with the blood NC and has higher sensitivity than the latter

To verify whether the increase in blood HBP level is closely related to neutrophil activation, we compared the relationship between blood HBP level and the peripheral blood NC. Firstly, we conducted a simple correlation analysis in 70 patients with autoimmune nephropathy to find the indicators correlated with the peripheral blood NC. As shown in Table 3, blood HBP level, blood CRP level, blood globulin level and peripheral blood platelet count were correlated with the peripheral blood NC (blood NGAL level was not correlated with the blood NC). In the partial correlation analysis, the blood NC was only correlated with blood HBP level and blood CRP level. Among them, the correlation between blood HBP level and blood NC (*R* = 0.466) was slightly stronger than that between blood CRP level and blood NC (*R* = 0.443).

**Table 3:** Correlation between neutrophil count and different inflammatory indicators.

Factors	Single-correlation			Partial-correlation		
	*R* value	95% CI	*p*-value	*R* value	95% CI	*p*-value
Serum HBP	0.381	0.123~0.581	0.002	0.466	0.281~0.677	<0.001
Serum CRP	0.325	0.085~0.539	0.009	0.443	0.044~0.685	<0.001
Serum globulin	0.283	0.012~0.499	0.020	0.085	−0.245~0.425	0.516
Peripheral platelet	0.281	0.017~0.508	0.024	0.147	−0.165~0.419	0.263

HBP, Heparin-binding protein; CRP, C-reactive protein; CI, confidence interval. *p*-value <0.05 was considered statistically significant.

**Figure 4:**
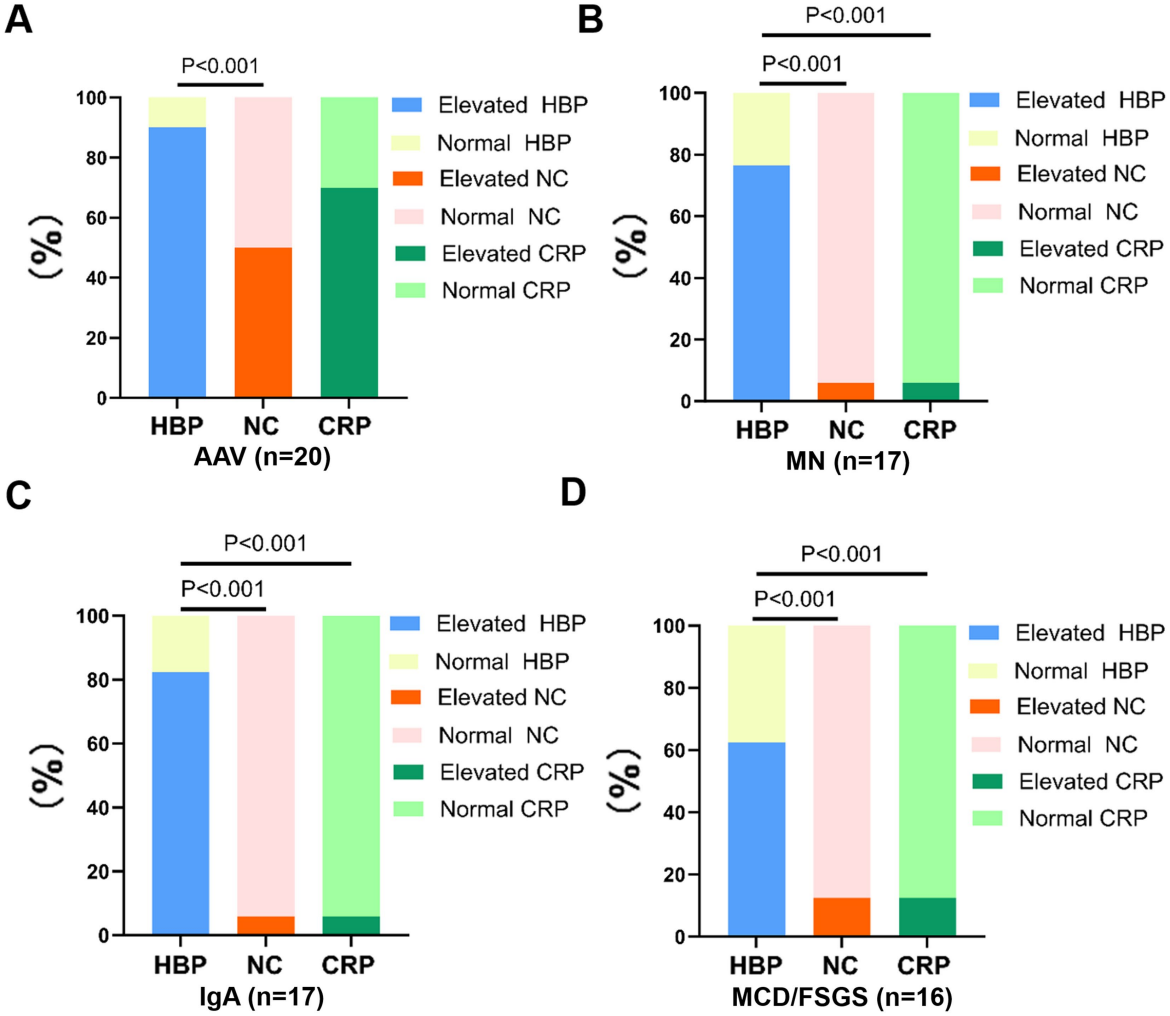
The proportions of elevated blood HBP, elevated peripheral blood neutrophil count (NC) and elevated blood CRP were compared, respectively, among patients with AAV **(A)**, MN **(B)**, IgAN **(C)**, and MCD/FSGS **(D)**.

We then compared the differences in the percentages of elevated blood HBP, NC, and CRP in these different autoimmune kidney diseases. The results are shown in Figure 4. In AAV, the percentage of elevated HBP was greater than that of NC and CRP, but only the difference between HBP and NC was statistically significant (*p* < 0.001), while the difference between HBP and CRP was not statistically significant. In MN, IgAN and MCD/FSGS, the percentages of elevated HBP were greater than that of NC and CRP, and the differences were all statistically significant (*p* < 0.001).

### 3.5 Analysis of factors influencing blood HBP levels in autoimmune kidney diseases via multivariable linear regression models

To further eliminate the influence of factors such as age and renal function on the blood HBP level in autoimmune kidney diseases, we conducted an analysis using a multivariable linear regression model. As eGFR was found to have significant collinearity with age (age is included in the eGFR calculation formula), we used serum creatinine level to replace eGFR. Four variables were included in the model: age, serum creatinine, NC, and blood CRP level. The model calculation formula is: Blood HBP = 6.817 + 0.300 × age + 0.020 × serum creatinine + 2.947 × NC + 1.072 × CRP. The adjusted *R*-squared of the model is 0.235, and the ANOVA results show that the *F* value of the model is 6.304, with a significance *p* < 0.001. The maximum condition index of the model is 9.682, and there is no need to handle multicollinearity issues. Among the four independent variables, only the NC has a significant independent positive predictive effect on the blood HBP (*B* = 2.947, *t* = 2.252, *p* = 0.028).

### 3.6 The clinical significance of elevated blood HBP in different autoimmune kidney diseases

**Figure 5:**
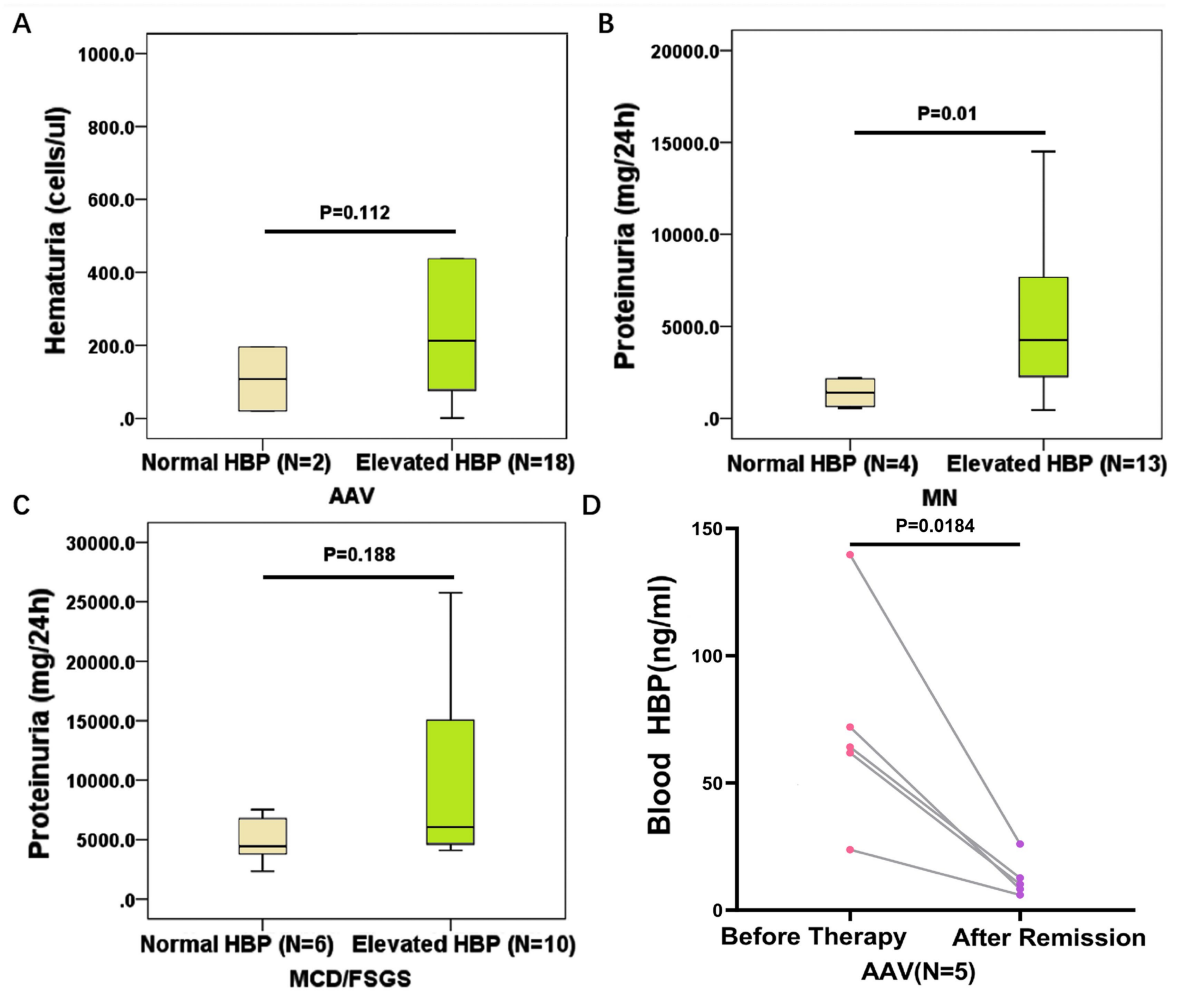
The clinical significance of elevated blood HBP in different kinds of autoimmune kidney diseases. **(A)** Comparison of hematuria levels between AAV patients with normal blood HBP and elevated blood HBP. **(B)** Comparison of proteinuria levels between MN patients with normal blood HBP and elevated blood HBP. **(C)** Comparison of proteinuria levels between MCD/FSGS patients with normal blood HBP and elevated blood HBP. **(D)** The blood HBP levels of 5 AAV patients with elevated HBP at the onset of the disease significantly decreased after achieving remission.

We further analyzed the relationship between blood HBP levels and kidney injury in different kinds of autoimmune kidney diseases. No association was found between blood HBP levels and the degree of renal function decline in all four kinds of diseases. In AAV, there was no difference in proteinuria levels between those with elevated blood HBP levels and those with normal blood HBP levels. The former had a higher trend in urine red blood cell levels, but the difference did not reach statistical significance (Figure 5A). In MN, those with elevated blood HBP levels had higher proteinuria levels than those with normal blood HBP levels, and the difference was statistically significant (Figure 5B). In MCD/FSGS, those with elevated blood HBP levels had a higher trend in proteinuria levels than those with normal blood HBP levels, but the difference did not reach statistical significance (Figure 5C). No association was found between blood HBP levels and proteinuria levels or urine red blood cell levels in IgAN.

To determine whether the level of blood HBP varies with disease status, we selected 5 AAV patients who had high blood HBP levels before therapy. After these patients received treatment and achieved clinical remission, the blood HBP levels were significantly reduced to normal range (*p* < 0.05, Figure 5D).

### 3.7 The relationship between blood HBP and urinary HBP in autoimmune kidney diseases

**Figure 6:**
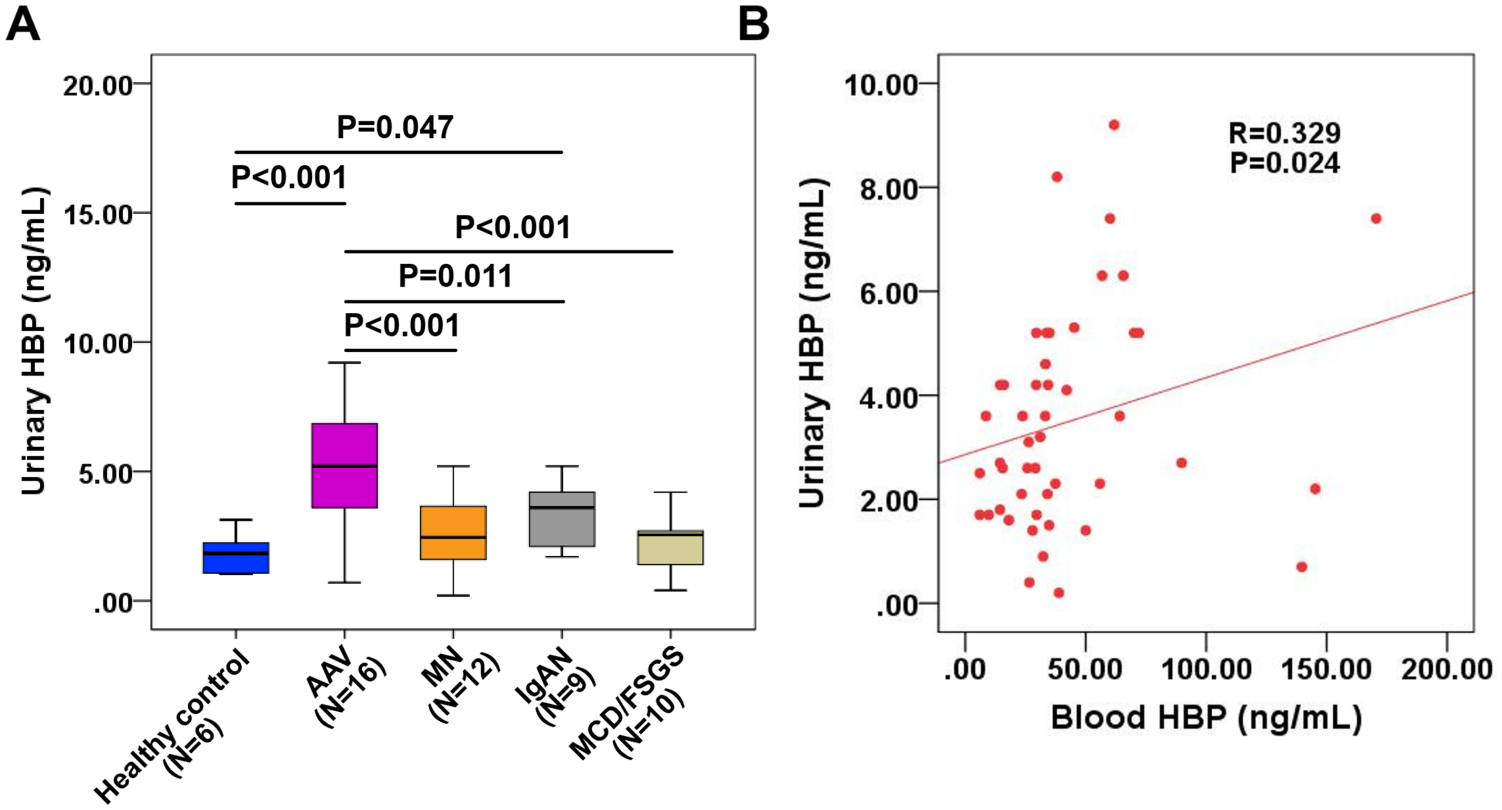
Measurement of urinary HBP in patients with autoimmune kidney diseases and its relationship with blood HBP. **(A)** Comparison of urinary HBP levels between patients with different kinds of autoimmune kidney diseases and healthy controls. **(B)** Correlation analysis of blood HBP and urinary HBP in 47 patients with autoimmune kidney diseases.

Among 70 patients with autoimmune nephropathy, 47 had available urine specimens for detection. Thus, we measured urinary HBP using an ELISA kit, with urine samples from 6 healthy individuals as controls. As shown in Figure 6A, the urine HBP level of AAV patients was significantly higher than that of the normal control group. The difference in urinary HBP level between IgAN patients and the normal control group was also statistically significant (*p* = 0.047). However, no significant difference was observed in the urinary HBP level between MN, MCD/FSGS patients and the normal control group. Correlation analysis of blood and urinary HBP in the 47 patients revealed a moderate positive correlation (Figure 6B).

## 4 Discussion

The significance of blood HBP elevation in infectious diseases has been extensively investigated. In a variety of bacterial infections, elevated HBP levels can be detected up to 72 h prior to clinical deterioration. In sepsis, HBP secretion occurs before the onset of hemodynamic instability and organ dysfunction, and precedes the release of other inflammatory biomarkers, including procalcitonin, CRP, tumor necrosis factor-α, interleukin-6, and interleukin-8. Thus, HBP exhibits certain advantages over conventional biomarkers in the context of infectious diseases. To our knowledge, this study is the first to apply blood HBP detection in autoimmune kidney diseases. The results of the study suggest that the activation of neutrophils seems to be a common phenomenon in autoimmune kidney diseases. The cytoplasm of neutrophils contains three types of granules and one type of secretory vesicle (10). Primary granules, also known as azurophilic granules, contain MPO, lysozyme (Lys), elastase (NE), defensins (DEF). HBP is also mainly stored in primary granules. Secondary granules, also known as specific granules, mainly contain lactoferrin (LF), Lys, NADPH oxidase, etc., and are also the storage sites of NGAL. Tertiary granules mainly contain gelatinases with matrix-degrading properties, which assist neutrophils in penetrating tissues. Secretory vesicles are structurally different from traditional tertiary granules and are formed by invagination from the cell membrane (11). In terms of the ease of activation, primary granules are the most difficult to activate, followed by secondary granules, then tertiary granules, and secretory vesicles are the easiest to be activated, fusing with the cell membrane under extremely mild stimulation and integrating their membrane proteins and contents into the cell membrane or releasing them (12). Theoretically, HBP, which is mainly stored in primary granules, should be more difficult to be released from the cell than NGAL, which is stored in secondary granules. However, existing studies have shown that during the development of neutrophils to the mature stage, some HBP in primary granules is targeted into secretory vesicles. Therefore, when cells are stimulated, HBP is rapidly released outside the cell along with secretory vesicles (13). Unlike HBP, the storage site of MPO is limited to primary granules. In this study, we found that the blood MPO levels of patients with MN, IGAN and MCD/FSGS were not higher than those of normal people, indicating that the activation degree of neutrophils in these diseases was weaker than that in AAV. Therefore, compared with MPO, HBP is a more sensitive indicator for evaluating the activation of neutrophils. Currently, there is no evidence that NGAL exists in secretory vesicles, so its release also requires stronger stimulation and a longer response time.

The AAV is currently recognized as an autoimmune disease caused by the activation of neutrophils (14). In the current study, the blood HBP levels of AAV were higher than those of MN, IgAN, and MCD/FSGS. Although the blood NGAL levels of AAV were also higher than those of the other three groups of autoimmune kidney diseases (data not shown), since most AAV patients had eGFR <60 mL/min, the blood NGAL levels of AAV patients did not fully represent the degree of neutrophil activation (15). In fact, among 49 patients with autoimmune kidney diseases and eGFR ≥60 mL/min, 36 had elevated blood HBP but only 3 had elevated blood NGAL, suggesting that although most neutrophils in patients with autoimmune kidney diseases are activated, the degree of activation may not be very high. In contrast, in patients with sepsis, even without renal function impairment, significant elevations in blood HBP and blood NGAL can be detected. Therefore, for patients with autoimmune kidney diseases including AAV, the degree of neutrophil activation is significantly lower than that in patients with sepsis.

MN is considered an autoimmune disease mediated by autoantibodies and dependent on complement activation, with neutrophils not being the main role. However, recent studies have found that neutrophil activation may exist in MN. A retrospective study involving 561 patients with idiopathic MN showed that an elevated neutrophil-to-lymphocyte ratio (NLR) was an independent risk factor for persistent proteinuria after treatment (16). A large-scale retrospective analysis of MN patients’ cohorts investigated the relationship between neutrophils and venous thromboembolism (VTEs). The results showed that the peripheral blood neutrophil count of MN patients with VTEs was significantly higher than that of patients without VTEs. In multivariate logistic regression analysis, neutrophils were an independent risk factor for VTEs in MN patients. Compared with the healthy control group, the plasma cfDNA levels in MN patients, especially those with VTEs, were significantly elevated, suggesting the possible existence of NETs derived from neutrophils in MN patients (17). A single-cell transcriptome study of 5 untreated primary MN patients further confirmed the phenomenon of neutrophil activation in MN (18).

Traditionally, the research on the pathogenesis of IgAN has mainly focused on mucosal immune abnormalities, the production of galactose-deficient IgA1, the formation of anti-Gd-IgA1 autoantibodies, and the deposition of immune complexes in the glomerular mesangial area. However, in recent years, more and more evidence has shown that neutrophils, as innate immune cells, play a crucial “effector cell” role in the amplification of inflammation and tissue damage in IgAN. Studies have found that the blood NGAL level is related to the severity of renal interstitial injury in IgAN patients (19). Moreover, a high blood NGAL level at the time of renal biopsy can predict the progression of IgAN (20). A recent single-cell sequencing study revealed that NETosis is upregulated in IgAN patients and plays a key role in the pathogenesis of IgAN by promoting the release of inflammatory cytokines (21).

The pathogenesis of MCD/FSGS is unclear. T-cell dysfunction is considered the main cause of the disease, but in recent years, it has been found that anti-CD20 monoclonal antibody treatment is effective, and a considerable proportion of patients have positive anti-nephrin autoantibodies, confirming the importance of humoral immunity (22). However, little is known about the role of neutrophils in the pathogenesis. A retrospective study of 145 children with steroid-sensitive nephrotic syndrome (SSNS) (the majority of whom were MCD/FSGS) found that high NLR and high CRP were associated with an increased risk of recurrence, infection, and renal insufficiency within 1 year (23). Another study found that some children with nephrotic syndrome had high levels of immature granulocytes (IGs), which were positively correlated with the recurrence of nephrotic syndrome (24). IGs are neutrophils formed during the maturation process of hematopoietic stem cells in the bone marrow and are usually not released or detected in the peripheral blood of healthy individuals. However, they can enter the peripheral blood during infection or inflammation (25). This phenomenon suggests the existence of inflammation and neutrophil activation in MCD/FSGS. In this study, the HBP level in the MCD/FSGS group was higher than that in the normal control group, but it was the lowest among the four kinds of autoimmune kidney diseases, suggesting that the activation degree of neutrophils in MCD/FSGS may be lower than in the other three diseases.

The findings of this study indicate that neutrophil activation is prevalent in autoimmune kidney diseases, and thus the underlying mechanism of such activation warrants further investigation. Recent studies have highlighted the central role of mTOR in coordinating cellular metabolic responses and inflammatory pathways, which may be relevant to the activation states observed in autoimmune kidney diseases (26–28). Recent studies have found that the PI3K/AKT/mTOR axis plays a significant role in regulating the formation of NETs (29). Elevated IL-23 in various inflammatory and autoimmune diseases has the effect of inducing the activation of mTOR in neutrophils and promoting the production of IL-17 and IL-22 (30). Additionally, mTOR is also crucial for the chemotactic ability of neutrophils. Under the stimulation of chemotactic signals, neutrophils can release ATP, which in turn stimulates the P2Y2 receptor, and the P2Y2 receptor promotes mTOR signaling (31).

This study found that blood HBP is a more valuable marker of neutrophil activation than blood NGAL, NC, and CRP, and is not affected by eGFR. Due to the small sample size, we were unable to draw reliable conclusions about the clinical significance of elevated HBP in autoimmune kidney diseases. Preliminary results showed that elevated HBP was associated with high levels of proteinuria in MN and similar results were found in MCD/FSGS. In AAV, elevated HBP was found to be associated with hematuria weakly, while no clinical significance of elevated HBP was found in IgAN. Nonetheless, through the analysis of five AAV patients with blood HBP data during the follow-up period, we found that the degree of neutrophil activation is indeed correlated with the disease activity. In a small sample size analysis of 47 patients with autoimmune kidney disease, we also found a correlation between the levels of HBP in blood and urine, which further suggests that neutrophil activation may be involved in the pathogenesis of the disease. It is important to note that even if the correlation between blood HBP levels and kidney injury can be definitively established with an expanded sample size, cautious interpretation of the underlying mechanisms remains warranted. The main finding of this study lies in confirming the universal activation of neutrophils in various autoimmune kidney diseases through the measurement of blood HBP. However, the elevation of blood HBP levels alone cannot be used to distinguish different types of autoimmune kidney diseases. Further investigations are required to determine whether circulating HBP levels differ between autoimmune and non-autoimmune kidney diseases. Another critical question is whether circulating HBP directly contributes to the pathogenesis of kidney diseases. According to previous studies, HBP can stimulate a variety of immune cells, including macrophages, T lymphocytes, and neutrophils. Additionally, HBP functions as a monocyte-specific chemokine by directly activating C-C motif chemokine receptor 2 expressed on the surface of monocytes. This activation triggers monocyte recruitment, exacerbates inflammatory responses, and induces tissue damage (32). Furthermore, HBP binds to proteoglycans on the endothelial cell surface, which increases endothelial permeability and upregulates the expression of monocyte chemoattractant protein-1, intercellular adhesion molecule-1, vascular cell adhesion molecule-1, and E-selectin in endothelial cells, thereby enhancing monocyte-endothelial adhesion (33). Subsequent to this binding event, protein kinase C and Rho kinase are activated, facilitating calcium ion influx into cells and driving cytoskeletal reorganization, which ultimately results in increased endothelial permeability (34). Thus, the elevation of blood HBP is not merely indicative of its role as a biomarker; it also possesses the potential to directly exacerbate kidney injury.

## 5 Limitations

As a preliminary study, this research has several limitations: First, the sample size is relatively small, especially in the subgroup analysis of eGFR ≥60 mL/min, where the number of cases is too few, which may affect the statistical power. The accuracy of these findings requires further validation. Second, this is a cross-sectional study. Although paired data before and after treatment of AAV patients suggest the dynamic changes of HBP, a larger sample prospective cohort study is still needed to verify the value of HBP in predicting disease recurrence, treatment response and long-term prognosis. Third, this study only included four kinds of autoimmune kidney diseases, and it is not clear whether neutrophils are activated in other kinds of autoimmune kidney diseases. Fourth, this study only detected the level of HBP in peripheral blood and urine, and did not simultaneously detect the expression of HBP in kidney biopsy, making it impossible to directly analyze the impact of neutrophil activation on the kidneys. Finally, the heterogeneity of diseases is a problem that cannot be ignored. In this study, only four kinds of autoimmune kidney diseases were included, which is not a comprehensive coverage. Moreover, even within the same disease entity, the pathogenesis varies among different individuals. In this study, it is still not possible to answer to what extent neutrophils are involved in these diseases.

## 6 Conclusion

This study demonstrates that blood HBP is a highly valuable marker of neutrophil activation and is not affected by renal function. There is a prevalent neutrophil activation in autoimmune kidney diseases, with the highest activation in AAV and the lowest in MCD/FSGS, but the mechanism requires further investigation.

## Data Availability

The raw data supporting the conclusions of this article will be made available by the authors, without undue reservation.
